# The lasting influence of Ebola: a qualitative study of community-level behaviors, trust, and perceptions three years after the 2014-16 Ebola epidemic in Liberia

**DOI:** 10.1186/s12889-023-15559-1

**Published:** 2023-04-12

**Authors:** Ronan F. Arthur, Lily M. Horng, Amos F. Tandanpolie, John R. Gilstad, Lucy K. Tantum, Stephen P. Luby

**Affiliations:** 1 School of Medicine, Stanford University, Stanford, California, USA; 2 Armed Forces of Liberia, Monrovia, Liberia; 3 School of Medicine, Uniformed Services University of the Health Sciences, Bethesda, Maryland, USA

**Keywords:** Ebola Virus Disease, Liberia, Community trust, Social epidemic preparedness, Misinformation

## Abstract

The majority of disease transmission during the 2014-16 West Africa Ebola epidemic was driven by community-based behaviors that proved difficult to change in a social paradigm of misinformation, denial, and deep-seated distrust of government representatives and institutions. In Liberia, perceptions and beliefs about Ebola during and since the epidemic can provide insights useful to public health strategies aimed at improving community preparedness. In this 2018 study, we conducted nine focus groups with Liberians from three communities who experienced Ebola differently, to evaluate behaviors, attitudes, and trust during and after the epidemic. Focus group participants reported that some behaviors adopted during Ebola have persisted (e.g. handwashing and caretaking practices), while others have reverted (e.g. physical proximity and funeral customs); and reported ongoing distrust of the government and denial of the Ebola epidemic. These findings suggest that a lack of trust in the biomedical paradigm and government health institutions persists in Liberia. Future public health information campaigns may benefit from community engagement addressed at understanding beliefs and sources of trust and mistrust in the community to effect behavior change and improve community-level epidemic preparedness.

## Background

The majority of disease transmission during the 2014-16 Ebola epidemic in Liberia occurred in the home or in the public square and the general community [1]. This community transmission was driven by a reluctance to seek healthcare for those with signs and symptoms of Ebola and cultural burial practices that involved washing the bodies of the deceased [2, 3]. Although behavior change through community engagement was later recognized as critical to reducing transmission [4–6], the top-down intervention approach initially adopted by the Liberian government, including mandatory cremation and enforced quarantine, failed to engage community leadership and led to widespread fears and distrust of the healthcare system [7, 8].

High mortality in Ebola treatment units and an overwhelmed healthcare system that would sometimes turn suspected cases away may have contributed to the reluctance to seek healthcare or to utilize the telephone hotline set up to locate and collect suspected Ebola cases [9, 10]. In a 2015 qualitative study in Sierra Leone, respondents told facilitators that if they called the response team to collect their family member, they would never see them again [3]. In Liberia, Ebola treatment units were known as ‘death traps’ [11], and community members said the Ebola Task Force teams could take days to arrive [8]. The national cremation policy contributed to distrust of the government [9], and families of the deceased often bribed burial teams to retain the bodies of their loved ones [8].

Distrust of the government was commonplace [12], and alternative explanations for the disease and its origins circulated widely [13]. These beliefs included ideas that the government had manufactured the crisis in order to receive international monetary aid, that witchcraft was the cause of Ebola-like symptoms, and denial that the Ebola epidemic was real [9, 11, 14]. Denial may pose a significant obstacle to behavior change because those in denial of the crisis have low perceived threat appraisal (as in the Health Belief Model [15]). While beliefs, misinformation, and distrust in the context of Ebola were significant barriers to epidemic response and behavior change, these beliefs and perceptions should be considered in the historical context of colonial exploitation, political instability, and civil war in Liberia [7, 16]. Communities in Liberia may have deep-seated distrust of government authorities, given public frustration with government corruption allegations and scandals that have persisted through multiple administrations [17]. Furthermore, breakdowns in communication between authorities and contradictory messaging in early stages of the epidemic may have contributed to community distrust in authorities and skepticism about the epidemic’s origins [18].

Epidemic preparedness in Liberia following Ebola has generally centered around biomedical preparedness, institutional systems, and healthcare worker education [19]. In response to the epidemic in 2014, the National Public Health Institute of Liberia was established to prevent and combat public health threats through improving institutional strength and capacity [20]. These developments in the wake of the Ebola epidemic represent a meaningful step forward in health system strengthening in Liberia and have led to stronger institutional preparedness and response to the COVID-19 pandemic. However, standard behaviors and norms within communities (e.g. burial practices and care taking of the sick) can become dangerous in the context of a high-mortality epidemic. Mechanisms for behavior change recommendations using trusted channels are important to establish before an emerging public health crisis, rather than during one. Thus, to prevent and control future outbreaks of infectious disease in Liberia, the social aspects of epidemic preparedness, including community-level trust, perspectives, and behaviors, should also be studied and supported. Social factors that influence preparedness for epidemics at the community level may include modes of social interaction within a community, ability and willingness to comply with epidemic control measures, and access to information and misinformation [21]. Even if biomedical preparedness structures are in place, community norms around health-seeking behavior and trust in health authorities will influence the effectiveness of epidemic response or prevention efforts.

This study is part of a large mixed methods investigation of perceptions, beliefs, and behaviors during and since the Ebola epidemic in Liberia. In a previously published large-N household survey conducted in three Liberian communities with different Ebola experiences [13], our team found that trust decreased during the peak of the epidemic in the most Ebola-affected region, indicating that trust may be most compromised when it is most needed. In the present qualitative study, we conducted nine focus group discussions in the same three communities. We asked participants to recount their experiences throughout the 2014-16 Ebola epidemic, from their perceptions and behaviors in the early stages of the crisis to the present day. We asked about beliefs in Ebola then and now, knowledge of the causes of Ebola, trust in government representatives and international non-governmental organizations (iNGOs) during and after the epidemic, and behaviors and perceptions that were or were not changed or adopted throughout and since the crisis.

## Methods

### Study design

During January and February 2018, after the 2014-16 Ebola epidemic and before the onset of the COVID-19 pandemic in 2020, we conducted nine focus group discussions in three Liberian communities with different experiences during the Ebola epidemic. We utilized a qualitative study design as we were interested in detailed, first-hand accounts of how and why beliefs, attitudes, and trust did or did not change during and since the epidemic. Focus group discussions were selected over other forms of qualitative data collection to leverage advantages of social interaction, including brainstorming, fact-checking, and disagreement [22]. The research team anticipated that rumors (i.e. hearsay), diverse beliefs, and disagreement might surface in discussion. Focus groups would allow these topics to be discussed by an interacting group, rather than in individual interviews, so that reactions to the ideas of others could be noted.

### Study sites

We purposively selected three Liberian locations (as previously described [13]) for their distinct experiences during the Ebola epidemic: Duazon in Margibi County with a high level of exposure to Ebola response activities and low incidence of Ebola; Careysburg in Montserrado County with low exposure to response activities and low Ebola incidence; and Tubmanburg in Bomi County with high exposure to response activities and high Ebola incidence. In each of these study sites, we partnered with local leadership to first conduct pre-mobilization meetings to notify the community.

Duazon is a town on the main road between Roberts International Airport and Monrovia and between JFK Memorial Hospital and the crematoria used during the Ebola epidemic. While Duazon had no active cases within the community, there was significant exposure to the activity of the epidemic, from burial and cremation teams to ambulances and the transportation of international aid workers. Careysburg did not have active cases within the city limits. According to the mayor, the city enacted a self-quarantine, where no persons were allowed in or out of the community during the peak months of the epidemic. Tubmanburg, an area subject to heavy fighting in the Liberian civil wars in the 1990s and early 2000s [23], had up to 250 suspected or confirmed cases by January 2015, and was the site of the first U.S.-built Ebola treatment unit in Liberia [24].

### Focus group enrollment and protocol

One discussion took place with each of the following groups: adult women, adult men, and male and female youths (youth was defined as age 18-35 per community norms). We recruited focus group participants purposively in central public areas identified by local leadership. Participants were screened for gender balance in the youth group, a wide age distribution in all groups, and a lack of personal relationships between participants in the same groups.

Two experienced Liberian focus group discussion moderators, one male and one female, led the focus group discussions. They were trained on a customized script, a document designed by the research team that standardized the way the study was introduced to participants, including ground rules and the sequence of questions and topics to cover during the discussion. This training included two iterations of pilot focus group discussions with Liberian participants in Sinkor, Monrovia. Feedback from these pilot focus groups informed iterative development of the semi-structured script, including the order and framing of discussion topics.

Focus groups were made up of eight individuals, lasted about 90 minutes, and took place indoors near community centers of each study site. Each participant was given a numerical identifier from one to eight. Participants were asked to raise their number when they wished to contribute or respond. The moderator would call them by number and the respondent would state their number again before speaking. This procedure allowed each participant to fully have the floor while expressing their thoughts, ensured anonymity by not using names as identifiers, and also served as a means to attribute participant numbers to each quote when referring to the transcript produced from the audio recording.

### Ethics

All methods were carried out in accordance with relevant guidelines and regulations. Local approval was sought and obtained from local leadership in all three study sites. Focus group discussion enrollees participated in a written informed consent process where the study purpose, their anonymity, audio-recording, and their right to drop out at any time were explained. Ethical approval was obtained from institutional review boards at the University of Liberia (Protocol no. 17-11-083) and Stanford University (Protocol no. 34436).

We note that the social science literature on Ebola often refers to community perceptions, ideas, and explanations as ‘beliefs’. For clarity and consistency, we will use this term in our manuscript as well, though we recognize it may sometimes imply a judgement of inferior veracity compared to conflicting biomedical ideas. Here, we use ‘beliefs’ to mean anything that people believe or think about their health and what they should do to improve their health [25]. We do not seek to cast judgement, but we also recognize that some beliefs are preferred from an epidemiological standpoint when trying to effect behavior change (e.g. believing Ebola is real versus believing it is not real).

### Analysis

The audio recordings were transcribed verbatim by trained Liberian researchers and analyzed using NVivo 12 software. The data were analyzed using a constant comparison analysis, which includes three primary steps: open coding, labeling small sections of the transcript; axial coding, grouping these labels into categories; and selective coding, developing themes and patterns expressed in each category [26, 27]. To establish reliability of the codebook, two researchers reviewed codes separately, identified any discrepancies in data interpretation, and reached an agreement on code definitions. Here we report themes established from this process and include direct quotes to illustrate the ideas expressed around each theme.

## Results

Nine focus groups - three groups each of eight adult men, eight adult women, and eight young adults - were assembled in Careysburg, Tubmanburg, and Duazon in Liberia. In total 72 participants were included; 51% were female (Table 1). Their average age was 39, and the average number of children was 3.8. Looking at the proportion of participants with ‘no education’ and ‘primary’ education in each study site (0.71 in Duazon, 0.42 in Carysburg, and 0.3 in Tubmanburg), participants in Duazon had a lower educational level than participants in each of the other two sites (as evaluated pairwise with 2-proportion z-tests; p<0.05). Emerging thematic topics from these conversations fell into three broad categories: behavior and behavior change in response to the epidemic, trust in the national government and in iNGOs, and beliefs about the causes and reality of Ebola (Table 2).

**Table 1:** Demographics of study participants

Characteristics	Overall	Duazon	Careysburg	Tubmanburg
Participants	72	24	24	24
**Sex**				
Male	35 (49%)	12 (50%)	12 (50%)	11 (46%)
Female	37 (51%)	12 (50%)	12 (50%)	13 (54%)
**Age**				
18-29	24 (33%)	9 (38%)	8 (33%)	7 (29%)
30-39	17 (24%)	7 (29%)	4 (17%)	6 (25%)
40-49	14 (19%)	4 (17%)	6 (25%)	4 (17%)
50+	17 (24%)	4 (17%)	6 (25%)	7 (29%)
**Education**				
No education	13 (18%)	6 (25%)	4 (17%)	3 (13%)
Primary	21 (29%)	11 (46%)	6 (25%)	4 (17%)
Secondary	28 (39%)	4 (17%)	12 (50%)	12 (50%)
Higher education	10 (14%)	3 (13%)	2 (8%)	5 (21%)
**Number of children**				
**per study participant**				
0	7 (10%)	2 (8%)	3 (13%)	2 (8%)
1-2	23 (32%)	8 (33%)	7 (29%)	8 (33%)
3-4	18 (25%)	7 (29%)	5 (21%)	6 (25%)
5+	24 (33%)	7 (29%)	9 (38%)	8 (33%)

**Table 2:** Emerging themes and key quotes on Ebola-related behaviors, trust, and beliefs from focus group discussion participants in Liberia, 2018

Topics	Emerging themes	Participant viewpoints	Key quotes
	Handwashing	Handwashing behaviors changed during Ebola and remain changed in 2018	Like before some people never had the idea of washing hands, even if you change your baby diaper or use the bathroom or something... But Ebola came everybody got the message. -Tubmanburg Women, R1
Behavior change	Social distancing	Social distancing significant during Ebola but had reversed by 2018	During the Ebola time when you go to the mosque you can’t sit down beside one another, no shaking hands. But now... you shake everybody hand. -Tubmanburg Women, R3
	Burial practices	Cultural burial practices were stopped during Ebola but had mostly reversed by 2018	Ebola people they come take you they go chuck you away... but now, now they bathing body. -Tubmanburg Women, R3
	Healthcare seeking	Would not call the Ebola hotline, perceived as fatal to do so	If you call them they come carry your patients never see that patient again. -Careysburg Youth, R1
	Government trust	Trust decreased during Ebola due to perceived incompetent management and corruption	Before the Ebola, I trusted the government fully, but when Ebola hit I lose confidence in the government. -Duazon Men, R5
Community trust	iNGO trust	iNGO workers were more trustworthy due to perceived benevolent incentives	I trusted the International partners that came here during the crisis time because they helped us. If somebody can put their life on the line for you, they should be commended for that. So they had no business coming, but... they volunteer to come and they came and help us. -Careysburg Men, R1
	Is Ebola real?	Personal experience with Ebola led to greater degree of belief	They took my auntie’s son, 24 year old man, the very next morning he pass away. So I say if my auntie’s son can die then we need to take Ebola business serious from there. -Duazon Youth, R8
Beliefs	Causes of Ebola	Ebola causal explanations in 2018 include: scientific understanding, a manmade virus, dirty water, and witchcraft	What I saw during the Ebola time is, the virus is a manmade virus, is not natural. They put it in the atmosphere to generate more fund. -Careysburg Youth, R4
	Other beliefs	Preventive measures were believed to help that did not agree with scientific understanding	If you drink cane juice [rum] you know Ebola can’t affect you. That you know right there I start drinking you know from there I started going into the cane juice shop. -Tubmanburg Youth, R3

### Behavior and behavior change

Many participants reported that handwashing behaviors underwent widespread change during the Ebola epidemic. Before the Ebola epidemic, they said, handwashing was not a common practice: ‘Before the Ebola... we can’t wash our hands before eating some of us we not know the thing... So I think it teach us some things’ (Careysburg Women). Many focus group participants reported that changes to handwashing behaviors were still in place in January 2018: ‘For me I wash my hand anything I do I wash my hand because it just used to me now’ (Careysburg Youth).

There were reports in all three communities that intervention efforts from iNGOs and health authorities included the provision of handwashing buckets and instructions for handwashing protocol. Community members were told to keep the buckets full of chlorinated or bleached water next to their door and have everyone wash their hands before entering the home. Many reported that, up to the present day, households and store owners had chosen to continue using the chlorinated buckets of water that had been distributed during the epidemic: ‘Like washing hands, Liberians were not use to washing hand, but nowadays you go to most homes you still find bucket sitting down on the porch’ (Careysburg Men).

**Key Quote:** The Town Chief bought a bucket for people to wash their hands in the meantime. Then Town chief make announcement around the Town that everybody should buy bucket to put water inside to wash their hands. Everybody was doing it on a daily basis, so [Ebola] never reach here because we were taking precaution. - Duazon Men

Participants in all three communities described quarantines, the closure of schools and markets, and travel restrictions during the Ebola epidemic as a hardship. ‘The economic crisis of our country was high. Our people was not able to go to market places to get their daily bread there was no money floating in our country’ (Tubmanburg Youth). Participants recalled that many could not work and could not buy food, so there was hunger and isolation.

In public, participants said, people would not shake hands or touch one another and would call less frequently on their friends and family. They would sit at a distance from one another in public: ‘I can remember in the church and all you go there people can say don’t sit down near me’ (Tubmanburg Women). By 2018, these social distancing behaviors and restrictions had more or less returned to pre-epidemic norms: ‘People used to be afraid to shake hands, people used to be afraid to hug one another but after everything we get back to normal thing. Only few people still going by it’ (Duazon Women).

One Careysburg participant explained the procedure for safe burial practices during the epidemic:

**Key Quote:** When somebody die the people say y’all should wait and call the burial team, they come and inspect the body before turning it over. If that Ebola then well they will carry it and do the burial, but if you call, if they check the body out if they see nothing like Ebola then well maybe they will turn the body over to the community people. - Careysburg Men

During the epidemic, not everyone obeyed the prohibitions on bathing bodies. One respondent in Tubmanburg witnessed these burial practices being carried out in secret: ‘Ebola time we saw people bathing body. My very self I went to go look for banana to fix my rice bread we bumped up with the people bathing the body in the banana bush’ (Tubmanburg Women).

In all three communities, the practices of bathing and dressing bodies, providing food at a funeral, and open caskets largely ceased during the epidemic. Focus group participants reported that these behaviors had mostly returned to pre-epidemic standards by 2018, though some reported that people were taking more care not to touch the bodies without gloves and to take precautions: ‘Since this Ebola business come the way it has gone, anybody dies we can’t just rush there to touch that body yet’ (Duazon Women).

Participants were divided in whether or not they called or would have called the Ebola hotline for help with handling the sick or deceased. A few participants said they would have done so or actually did: ‘My big brother woman... got infected from Ebola, from there you know we stop going closer to her. The first and foremost thing I did is that I called you know at that time we were using the 4455’ (Tubmanburg Youth).

In all three communities, however, it was more commonly reported that calling the hotline was not an acceptable option because the sick person they were trying to help would never come back if they were taken away by authorities. Other behaviors that were adopted or changed for preventive reasons following public health recommendations included wearing masks, long sleeves, and gloves, the latter for touching the sick or their fluids. ‘You wear long sleeve and put thing some people can put something on their nose just like doctor’ (Careysburg Women). Participants reported that behaviors and practices to clean up after the sick were still practiced in 2018.

**Key Quote:** That time my son stomach was running they say that Ebola was on my son. They… wanted to carry my son. They… wanted call the [Ebola hotline] people. I cry on them I say my people I beg you’re the thing that happening to my son that not Ebola - the little boy skin just hot, he vomiting, toileting, that not Ebola. That’s time I went to the town chief. He talked to them for me. That how they leave it, they never called Ebola. - Duazon Youth

### Community trust

The degree of trust in the government varied among participants and led to disagreement at times.

First respondent: *I felt the government was good because Ebola came... they taught us how to take care of ourselves.*

Second respondent: *Maybe the government help that in their own community but for us this here government never did nothing.*

- Duazon Youth

Participants who said they trusted the government commonly pointed out that the epidemic could have been worse. Others supported the government’s role during the epidemic and pointed to the government cooperation and collaboration with foreign aid efforts that brought material relief: ‘NGO, government they, both of them, worked equal in the process because the fact of the reality the government allowed them to come in the country to help the citizen’ (Tubmanburg Youth). However, most participants distrusted the government and felt it had not responded properly to the epidemic. Justifications given in all three communities to support this viewpoint were incompetence, corruption, and mismanagement on the part of the government. One participant speculated that if the epidemic had happened in another part of the world, the government there would have more competently contained and solved the problem.

Most participants agreed that Ebola relief money was not well spent by the government. One participant cited widespread food and hunger problems that were not addressed: ‘They should have bought rice... and share it on us’ (Duazon Youth). Many participants claimed that government officials were pocketing Ebola relief money for themselves. There was suspicion that the government was perversely incentivized to prolong the crisis due to the relief money that was coming in. It was reported that the money for Ebola relief and survivors never made it to the people it had been intended for due to corruption: ‘They say reach the money out to the layman on the field... When they come the money will stop on the half way’ (Careysburg Men).

**Key Quote:** In this country people dying then you taking money from different countries... putting it in your pocket, not seeking the people interest. Some people got rich out of this sickness, so I not trust them. - Duazon Men

In contrast to trust in government, reported levels of trust in international relief organizations were consistently high: ‘The international NGO, yeah, I hundred percent trust them’ (Tubmanburg Youth). Many participants reported that, while the government had performed incompetently during the epidemic, the international organizations had performed well and were trustworthy: ‘It is the NGO that did extremely well for the people of Liberia, because the government herself don’t have no system when it comes to health’ (Tubmanburg Youth). Aid and relief from international NGOs brought up in discussion included information dissemination, handwashing equipment, food, and infrastructure: ‘This Ebola bucket they was sharing it and they was having some NGO come in start providing food for people so they did well’ (Tubmanburg Women).

**Key Quote:** The government it was not one day for me to see any government officials coming in the various community to carry on sensitization, except when NGO is going you will see one with that NGO. - Tubmanburg Youth

Participants commonly credited the iNGOs with hands-on relief efforts, including direct representation in communities: ‘I say thank you because the NGOs their self were coming. If they had placed the money in government hand… then by this time we still in this Ebola problem’ (Careysburg Men). In discussions of trust, iNGOs benefited from perceived benevolent intentions. While the government was seen as motivated to receive relief money and not to stop the epidemic, iNGOs fell under less suspicion because of visible relief efforts, but also because they did not appear to have ulterior motives during the crisis.

### Beliefs

Nearly three years after the epidemic, there was still disagreement in most of the focus groups about whether or not Ebola was real. It was reportedly very common to not take the disease seriously in the early days of the crisis, to joke about it or be skeptical about the veracity of the information received: ‘Nothing changed in my behavior, I used to go out normally, shake people hands, joke with people until the whole thing was over. Because as for me I believe that Ebola was not real’ (Duazon Women).

**Key Quote:** It was not really serious... I never used to believe it at all, I just took it to be something that they just wanted to use, you know, to make money. Until when Ebola enter into Bomi County and stretch out in the community. - Tubmanburg Youth

Personally witnessing the effects of Ebola was highly influential in changing beliefs about the reality of Ebola (as in the above key quote from the Tubmanburg Youth group), according to participants in all three communities. ‘They say seeing is believing, something that you never see before you will not believe it. That’s how I felt’ (Careysburg Youth). With family connections to infected and deceased individuals in neighboring communities, one woman in Duazon said she took Ebola seriously and believed it was real: ‘I take it to be real thing because my brother died, woman died everybody. Only on my own I was in this town here because all my family them died from Ebola’ (Duazon Women).

While many participants expressed the view that Ebola was real, there were respondents in all three communities that said they still did not believe it was real: ‘No even up to this time I don’t believe it’ (Duazon Women). This belief was supported through three lines of reasoning. The first was that Ebola was part of a government conspiracy to make money or deploy military forces. The second theme was an expression of disbelief if the respondent had not personally seen anyone infected: ‘This Ebola, Ebola me I not believe it because in our community Barnesville, you know... nobody died’ (Tubmanburg Women). A third reason repeated by multiple participants was that people still came down today with the same symptoms that had characterized Ebola, so the information about Ebola must be inaccurate.

There were a variety of opinions about the causes of Ebola, ranging from wild animals to scientists and from weather to witchcraft. When directly asked about causes of the epidemic, only four individuals in three of the nine focus groups expressed views in agreement with scientific research: ‘Ebola was caused by other wild animals like monkey, baboon, bat, and others and even plum when bat eat the plum, it drop you not supposed to eat it’ (Duazon Youth).

While a few participants mentioned that bushmeat, meat from wild animals, was a potential cause of Ebola, most participants reported that they did not believe this. They cited their own experiences of eating bushmeat without contracting Ebola as evidence against this transmission pathway and, in one case, for the reality of Ebola in general: ‘The Ebola that came, meat did not bring it. So we were still eating it in the bush, we been eating it. Ebola finished and it didn’t reach to us’ (Duazon Women). Participants generally reported that bushmeat was commonly eaten in 2018: ‘In my community people are still eating bush meat’ (Careysburg Youth).

In all three communities, some respondents reported that they believed the virus was manmade. In Duazon, there was a rumor that the Americans had invented Ebola in order to develop and test a weapon that they would unleash on their own minorities and that the Liberian government had agreed to the experiments: ‘They said that this sickness we want to know whether it can kill so we want to inject it in a human to know how long it will take before the person dies’ (Duazon women). Others reported that the Liberian government was responsible. They pointed to the monetary aid that resulted from the crisis as a motivation for allowing the disease in or even for directly spreading it.

Many people rejected the notion that Ebola was caused by witches, but there was some disagreement about this. There was widespread suspicion that food and water sources were poisoned with Ebola. These poisonings were sometimes attributed to witches, but were more often attributed to the government.

**Key Quote:** About the story about witchcraft... They say they were cooking the church people food, they went and poison the food... When they eat that food, every one of them died. Then put it there on Ebola. - Duazon Men

Beliefs about the causes of Ebola, preventive measures, and those responsible that do not agree with the scientific narrative still existed three years after the end of the epidemic and were reported in all focus group discussions. In Duazon, there was a persistent belief that all participants agreed on - the government hired people to ‘bust heads,’ physically destroy the heads of Ebola victims through blunt force before cremation: ‘The person already died then... somebody must go bust their head and you give them huge amount of money, they build house and they did lot of things and they let the Liberian people die’ (Duazon Women). This belief was only discussed in Duazon, a town near to the cremation sites the government used during the epidemic, but was discussed at length there.

Some behaviors and behavior changes were reported that came from alternative ideas about Ebola transmission pathways. One participant in Tubmanburg tried bathing in salt water: ‘I did it because you know for me to be safe from the Ebola that’s what they said, they said you know everybody must bathe with salt water, Ebola can’t come close to you. I did it we all that in our house we bathe with salt water just for Ebola.’ (Tubmanburg Youth). Another respondent in Tubmanburg reported that drinking alcohol could be preventive and began drinking to protect himself.

## Discussion

Results from these nine focus group discussions suggest that diverse communities in Liberia in the aftermath of the 2014-16 Ebola epidemic do not trust the government and do not, by and large, subscribe to the biomedical understanding of Ebola. While some behavior changes have persisted, including handwashing and safe practices in caring for the sick, other Ebola-related behaviors undertaken during the epidemic were widely reported to have reverted to prior standards, including social proximity, handshaking, and some burial practices. Such reversions to prior social behavior are not cause for alarm as communities would not be expected to retain these changes in the absence of a public health crisis. However, denial of the existence of Ebola, beliefs in non-biomedical causal mechanisms, and general distrust of government institutions have remained prevalent perceptions and sentiments in geographically and epidemiologically diverse Liberian communities. As trust, beliefs, and perceptions at the community-level are essential to timely and effective epidemic response, our findings suggest that Liberian communities may not be well prepared to respond quickly during the next outbreak of Ebola or of other infectious diseases.

Many respondents denied that the Ebola epidemic had ever been real. While it is understandable that in the chaos of the crisis there might be widespread skepticism and rejection of mainstream narratives, it is notable that this belief is still broadly held in communities with different Ebola epidemic experiences. Indeed, one qualitative study conducted in August-September 2014 found that residents of Monrovia were rapidly changing their minds about Ebola and discarding incorrect information [28]. However, our study indicates that belief in misinformation did not disappear as alternative causal mechanisms, deep distrust of the government, and denial were still prevalent elements of our focus group discussions held in 2018. Without common recognition of an epidemic, perceived threat is non-existent, and behavior change is not viewed as important or warranted [15]. Widespread denial reflects deep-seated distrust of the government held by many Liberians [12, 13] and represents an unsolved problem that is likely to re-emerge during future public health crises. This study provides insight into drivers of distrust and denial that continue to undermine community readiness for future epidemics in Liberia. To enhance community epidemic preparedness, it is essential to empower local leaders to participate in prevention and response efforts, address community member concerns and skepticism, and involve communities in decision-making around preparedness and response efforts [29].

Narratives and stated justifications on trust in the government and iNGOs suggest that participants perceived these two groups and their activities during the Ebola epidemic differently. Many participants expressed a high level of suspicion and distrust of the government, citing corrupt financial incentives of the government and asserting that the government initiated and intentionally prolonged the epidemic to receive aid funds. The focus on financial mismanagement and corruption as a point of greater contention than the enforced behaviors, quarantines, and top-down intervention approaches cited by the literature (e.g. [7–9]) is noteworthy. It may reflect the public perception of and concern with corruption and graft in the government that has been a forefront political issue for both Presidents Ellen Johnson Sirleaf and George Weah for nearly two decades. By contrast, iNGOs were widely viewed as highly trustworthy, a finding consistent with two distinct quantitative household surveys [12, 13], due to perceived benevolent intentions for their presence and intervention in Liberia. These findings further complicate the historical tension between iNGOs and weak state governments mired in allegations of corruption in how the humanitarian needs of the people should be addressed and who should address them. On the other hand, community trust in foreign organizations presents an opportunity to strengthen the long-term health system of the country by leveraging international support garnered by the crisis. Establishing trusted communication channels between government actors and communities, with the collaboration of iNGOs, could generate greater community resilience [30, 31]. Furthermore, governments and foreign organizations should collaborate with and support local NGOs and community-based organizations in epidemic response efforts. Local NGOs may have close ties with communities and therefore be well-positioned to understand and address the root causes of distrust.

Our qualitative results may add insights worthy of further empirical consideration and ultimately useful to mathematical modeling of epidemics. Epidemic modeling has not typically included behavior change within the classic compartmental modeling framework [32–34]. An early CDC Ebola model, for example, assumed no behavior change and projected a final epidemic size of 1.4M cases of Ebola in Liberia and Sierra Leone by January 2015, orders of magnitude more than the true final epidemic size [35]. This is because individuals and governments reacted to the risk posed by the epidemic, and we should expect such reactions. In adaptive behavior models, economists and modelers often assume that as prevalence increases, behavior improves, leading to a negative feedback loop that dampens epidemic dynamics as may partially explain the trajectory of the 2014-16 Ebola epidemic [36, 37]. Our focus group results corroborate this as several respondents reported changing their minds that Ebola was real after witnessing victims of the virus first-hand. However, trust was at its lowest point as the epidemic was at its peak in the high-incidence region, a finding supported quantitatively in the study’s parallel empirical research [13]. If trust is compromised as prevalence increases and trust affects behavior change [12], then distrust may produce a positive feedback loop working against the negative feedback of prevalence and behaviors assumed in most adaptive behavior models. Modelers and public health practitioners should thus consider these other important psychological factors that drive behavior and may have important consequences for epidemic dynamics.

Distrust and skepticism may lead to the rejection of the international biomedical paradigm and associated efforts from local governments to promote healthy behaviors both during and after public health crises. These beliefs and attitudes toward government are important to social preparedness as they affect the likelihood of behavior uptake that will avert lives lost to infectious disease. Several of the themes discussed in this manuscript have re-emerged in the context of COVID-19. For example, COVID-19 vaccine hesitancy is highly prevalent in Liberia; 66% of Liberians self-reported as unlikely to get vaccinated in a 2021 survey [38]. Distrust of vaccine development motivations from international and domestic leaders is an important driver of vaccine hesitancy [39]. Colonial history and previous abuses of medical and vaccine research may also be contributing to contemporary suspicions and skepticism about COVID-19 [40]. For example, a 15-country Africa CDC study found that 43% of those surveyed believed that Africans were being used as guinea pigs in vaccine development [41]. An early survey of COVID-19 vaccine acceptance found that those with higher levels of trust in government were more likely to get a vaccine [42]. In the Democratic Republic of Congo, in a large-n cross-sectional survey, 24% of respondents believed that COVID-19 was not real at all [43]. Between May 2022 and September 2022, the proportion of fully vaccinated individuals in Liberia rose from 28% to 57% [44], demonstrating that popular opinion of the vaccine shifted rapidly in Liberia, but with significant delays due to supply, distribution, and community perceptions. COVID-19 has demonstrated that community resilience and social epidemic preparedness are important issues beyond the Ebola crisis and merit significant attention to further understand and improve.

This study should be evaluated in the context of its limitations. While focus group discussions promote collective discussion and bring a variety of voices to the table, they may also inhibit extreme or fringe points of view relevant to the topic but that go unexpressed. Due to our sampling method, we may have not accessed individuals in isolation or otherwise not out in public centers, individuals whose views may have also been more divergent from popular opinion. While these perspectives would add nuance to our findings, our objectives were primarily focused on widely-held views that are commonly discussed in public. Focus group discussions are subject to social power dynamics; voices of the most powerful members of the group are more likely to be heard than voices of the less powerful. We organized our focus groups by age and gender to minimize these power dynamics, and our facilitators encouraged quiet respondents to answer first, though we acknowledge some higher status participants may still have voiced their opinions more frequently than lower status participants. The study asked participants to discuss events three years in the past, the memories of which are subject to degradation or influence from the narratives of others over time. While this may have impacted accuracy of memory, narratives about the Ebola epidemic told today, whether accurate or inaccurate, are relevant to the study of current perceptions and beliefs and will influence the actions of individuals in the event of a future crisis.

## Conclusions

Ebola has had a lasting influence on community perspectives in Liberia. Resilience to future public health emergencies depends on how communities react to them, but our findings suggest that the legacy of Ebola is not a unifying experience of increased trust, belief in biomedical principles, and trust of health authorities. Distrust of the government, trust of iNGOS, denial of Ebola, and perceived corrupt government incentives were found to be prevalent in all three communities. We stress that these problems are the responsibility of those in positions of authority to address and thus recommend further efforts to develop and evaluate public health interventions and conversations that improve trust in the government. Long-term investment in reducing government corruption, closer collaboration between the government and iNGOs for healthcare (as has been encouraged by the World Bank since the 1990s [45]), and the direct engagement of local leadership, such as of a town chief or religious leader, in communication campaigns may be effective strategies to improve community resilience to public health crises in Liberia [7, 30]. Public health communication campaigns should use anthropological research identifying specific lines of reasoning and beliefs that encourage denial and distrust and provide rational counterarguments through trusted sources [31]. We recommend research to identify best practices for trust-building and for communication via trusted sources during both times of crisis and times of relative stability. We do not need to wait until the next public health crisis to improve social epidemic preparedness in Liberia and in other low-income countries.

## Data Availability

The datasets generated and/or analysed during the current study are not publicly available due to privacy concerns but are available from the corresponding author on reasonable request.

## Abbreviations

CDC: Centers for Disease Control
COVID-19: Coronavirus disease 2019
iNGOs: International non-governmental organizations
